# Prospective Study of Non-Contrast, Abbreviated MRI for Hepatocellular Carcinoma Surveillance in Patients with Suboptimal Hepatic Visualisation on Ultrasound

**DOI:** 10.3390/cancers16152709

**Published:** 2024-07-30

**Authors:** Mathew Vithayathil, Maria Qurashi, Pedro Rente Vicente, Ali Alsafi, Mitesh Naik, Alison Graham, Shahid Khan, Heather Lewis, Ameet Dhar, Belinda Smith, Nowlan Selvapatt, Pinelopi Manousou, Lucia Possamai, Hooshang Izadi, Adrian Lim, Paul Tait, Rohini Sharma

**Affiliations:** 1 Department of Surgery and Cancer, Imperial College London, London W12 0NN, UK; mathew.vithayathil@doctors.org.uk (M.V.);; 2 Department of Brain Sciences, Imperial College London, London W12 0NN, UK; 3 Department of Interventional Radiology, Imperial College Healthcare NHS Trust, London W12 0HS, UK; 4 Department of Nuclear Medicine, Imperial College Healthcare NHS Trust, London W12 0HS, UK; m.naik@nhs.net; 5 Department of Hepatology, Imperial College Healthcare NHS Trust, London W12 0HS, UKameet.dhar1@nhs.net (A.D.); nowlan.selvapatt@nhs.net (N.S.);; 6 School of Engineering, Computing and Mathematics, Oxford Brookes University, Oxford OX3 0BP, UK; 7 Department of Radiology, Imperial College Healthcare NHS Trust, London W12 0HS, UK

**Keywords:** hepatocellular carcinoma, surveillance, magnetic resonance imaging, obesity

## Abstract

**Simple Summary:**

Patients with chronic liver disease and cirrhosis are at risk of developing liver cancer (HCC). Regular ultrasound screening for HCC is recommended for these patients so that HCC can be found early. However, ultrasound is not always effective at picking up small cancers, especially in patients who are overweight or obese. Other tests including CT and MRI are expensive. Our study looked at a shorter version of an MRI scan (abbreviated MRI, or aMRI) in thirty patients who had recently had an ultrasound with poor views of the liver. All thirty patients tolerated the aMRI scan well. In these patients, the aMRI scan found one HCC and five other liver abnormalities which had not been picked up on ultrasound. Experts evaluated the aMRI scans and felt they were of good quality. Our study shows that aMRI is possible and useful in patients undergoing screening for HCC, especially those who have had poor views on an ultrasound.

**Abstract:**

Background: Biannual ultrasound (US) is recommended for hepatocellular carcinoma (HCC) surveillance in patients with cirrhosis. However, US has limited sensitivity for early-stage HCC, particularly in overweight cohorts, where hepatic visualisation is often inadequate. Currently there are no robust imaging surveillance strategies in patients with inadequate US visualisation. We investigated the ability of non-contrast, abbreviated magnetic resonance imaging (aMRI) to adequately visualise the liver for HCC surveillance in patients with previously inadequate US. Methods: Patients undergoing US surveillance, where liver visualisation was inadequate (LI-RADS VIS-B and VIS-C), were prospectively recruited. Patients underwent non-contrast T2-weighted and diffusion-weighted aMRI. The images were reviewed and reported by an expert liver radiologist. Three independent, blinded radiologists assessed the aMRI visualisation quality using a binary score assessing five parameters (parenchymal definition, vascular definition, coverage of the liver, uniformity of liver appearance and signal-to-noise ratio). Results: Thirty patients completed the aMRI protocol. The majority (90%) had underlying cirrhosis and were overweight (93.3%), with 50% obese and 20% severely obese. A total of 93.3% of the aMRI scans were of satisfactory quality. Six patients (20%) had hepatic abnormalities detected with aMRI that were not seen on their US: one HCC, one haemangioma and three clinically insignificant lesions. For the aMRI visualisation quality assessment, the coverage of the liver, vascular definition and parenchymal definition were consistently rated to be of sufficient quality by all three radiologists. Conclusions: Non-contrast aMRI provided good visualisation of the liver and detection of abnormalities in patients with inadequate US. aMRI should be further explored in a larger, prospective study as an alternative surveillance strategy in patients with inadequate US.

## 1. Background

The incidence of hepatocellular carcinoma (HCC) is rising and is now the third most common cause of cancer-related death worldwide [1]. The majority of patients develop HCC on a background of chronic liver disease (CLD) [2]. Patients with chronic hepatitis B (CHB) and a high viral load also have an elevated risk of HCC, independent of liver fibrosis [3]. The presence of an at-risk population allows for targeted surveillance [2]. Six monthly ultrasounds (USs) form the backbone of HCC surveillance in patients with CLD and CHB and are recommended globally by professional organisations [2,4]. The goal of US surveillance is the early detection of HCC, for which curative therapies can be administered, improving patient outcomes [2]. Whilst US surveillance is cost effective [5] and readily available worldwide, surveillance has a number of limitations. The reported sensitivities for the detection of HCC by US surveillance vary greatly between 28% and 100% [6], reflecting, in part, the issue of operator dependence in characterising lesions [7]. Moreover, a recent meta-analysis reports a sensitivity of only 47% for the detection of early HCC [6]. Sensitivity is further hampered by liver steatosis, cirrhosis and body habitus, in particular obesity, a key issue given the increasing rates of obesity and non-alcoholic fatty liver disease (NAFLD) globally [8]. Additionally, some NAFLD patients are known to develop HCC prior to the development of cirrhosis. An estimated 20% of ultrasounds are suboptimal [9]. Whilst there is increasing recognition of the impact of body habitus on surveillance quality, imaging strategies have not been refined, and there are no evidence-based imaging guidelines for patients who have inadequate surveillance ultrasounds. The American Association for the Study of Liver Diseases [10] and the Korean liver cancer guidelines [11] recommend a diagnostic, dynamic contrast-enhanced (DCE) MRI or diagnostic CT in the setting of poor US visibility; these scans are expensive, time consuming, carry the risk of cumulative gadolinium and radiation toxicity and have not been validated for surveillance [6,12]. Some centres persist with US surveillance despite not being able to visualise the liver.

Abbreviated MRI (aMRI) has fewer sequences than a diagnostic MRI and can be administered with or without contrast. A number of studies have shown the improved diagnostic accuracy of aMRI techniques compared to US [13,14,15,16,17], leading to aMRI gaining traction as an alternate surveillance imaging tool. Currently, there is no overall consensus on which MRI sequences should be included or whether contrast should be administered. Findings from the analyses of retrospective cohort studies of MRI in patients with CLD highlight the importance of including diffusion-weighted imaging (DWI), as the restriction of fluid is a feature of malignancy [18,19], combined with T2-weighted imaging (T2WI). However, the utility of aMRI has not been prospectively assessed in patients where the liver cannot be clearly visualised with US. Recognising the limited MR capacity in most countries and the acceptability of US for the majority of patients, this study investigated the ability of non-contrast, abbreviated magnetic resonance imaging (aMRI) to adequately visualise the liver for HCC surveillance in patients with previously inadequate US visualisation.

## 2. Methods

### 2.1. Study Population

Adult (>18 years old) patients with known cirrhosis and/or CHB with a high viral load attending general hepatology clinics at Imperial College Healthcare NHS Trust, London, UK, were eligible. Cirrhosis was defined by radiological and/or histological evidence as previously described [20]. Patients undergoing 6 monthly HCC surveillance scans, where the liver was reported as not clearly visualised on the US, were prospectively enrolled. Inadequate US surveillance was defined according to the Liver Imaging Reporting and Data System (LI-RADS), whereby VIS-A is no or minimal limitations, VIS-B is moderate limitations and VIS-C is severe limitations [21]. Written informed consent was taken for all patients. Patients were invited for an aMRI at the Imperial College London Clinical Imaging Facility within 28 days of US surveillance. Exclusion criteria included patients with a previous or current diagnosis of HCC, patients ineligible for HCC therapy as per the Barcelona Clinic Liver Cancer (BCLC) staging system [22] (Child–Pugh class C; Eastern Cooperative Oncology Group (ECOG) performance status ≥ 3) and contra-indications to MRI (including presence of incompatible metallic foreign objects). Baseline characteristics for all patients were collected, including their age, sex, ethnicity, underlying liver disease aetiology, comorbidities, ECOG performance status, body mass index (BMI) and Child–Pugh class. Ethical approval was obtained from the Health Research Authorities (reference 198951).

### 2.2. aMRI Protocol

MRI data were acquired using a 3T Siemens Magnetom Verio (A Tim System) scanner (Siemens AG, Munich, Germany) with Siemens Software Version Syngo MR B17. Each patient was scanned in the supine position. The aMRI protocol was developed by an expert hepatobiliary radiologist (PT) and research radiography team (PRV) alongside an oncologist with imaging expertise (RS).

The protocol consisted of a localiser for planning, followed by a respiratory-triggered diffusion-weighted image (DWI; approximately 5 min) and a respiratory-triggered T2-weighted image (approximately 2 min). DWI and T2WI sequences were selected based on their efficacy from previously reported retrospective aMRI protocols [17,18,19]. Images were acquired with a field of view of 400 mm, covering the liver, and a 6 mm slice thickness to permit an adequate signal-to-noise ratio to favour pathology comparison and image co-registration. The duration of the aMRI was determined from the time of the first imaging sequence to the completion of the final sequence.

### 2.3. aMRI Reporting and Quality Scoring

**Figure 1:**
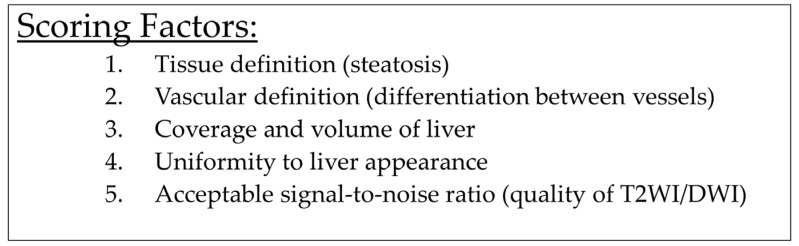
Visualisation quality parameters used to determine the quality of abbreviated MRI. Three independent, blinded radiologists assessed the visualisation quality parameters for each MRI. Each parameter was given a binary score of 1 for “acceptable” or 0 for “unacceptable”.

US quality was assessed by an US radiologist with over 25 years of experience (AL). aMRI scans were formally reported by a liver radiologist with over 40 years of experience (PT), and the overall ability of the aMRI to provide satisfactory HCC surveillance was assessed. In order to assess the visual quality of aMRI, a visualisation quality score was derived. Three independent radiologists (AA, MN and AG) who were blinded to the reports were asked to independently score the aMRI. As no validated scores exist that assess the quality of non-contrast aMRI for HCC surveillance, a novel visualisation quality score evaluating five parameters from each aMRI scan was proposed (Figure 1). This score was derived by an expert liver radiologist in conjunction with an expert liver oncologist with extensive imaging experience. The independent radiologists received training to assess each parameter in the aMRI. The parameters evaluated were parenchymal definition, vascular definition (ability to differentiate between vessels), coverage and volume of the liver seen on imaging, uniformity of liver appearance and signal-to-noise ratio. For each parameter, a score of 1 was given if the quality was sufficient, with a score of 0 given if inadequate.

### 2.4. Statistical Analysis

Inter-observer agreement for each aMRI visualisation quality parameter score between the three independent radiologists was calculated using Randolph’s free-marginal multirater kappa (κ) statistic [23]. All statistical analyses were performed using MedCalc for Windows, version 19.4 (MedCalc© Software, Ostend, Belgium), and Python 3.10.1.

## 3. Results

### 3.1. Study Population

**Figure 2:**
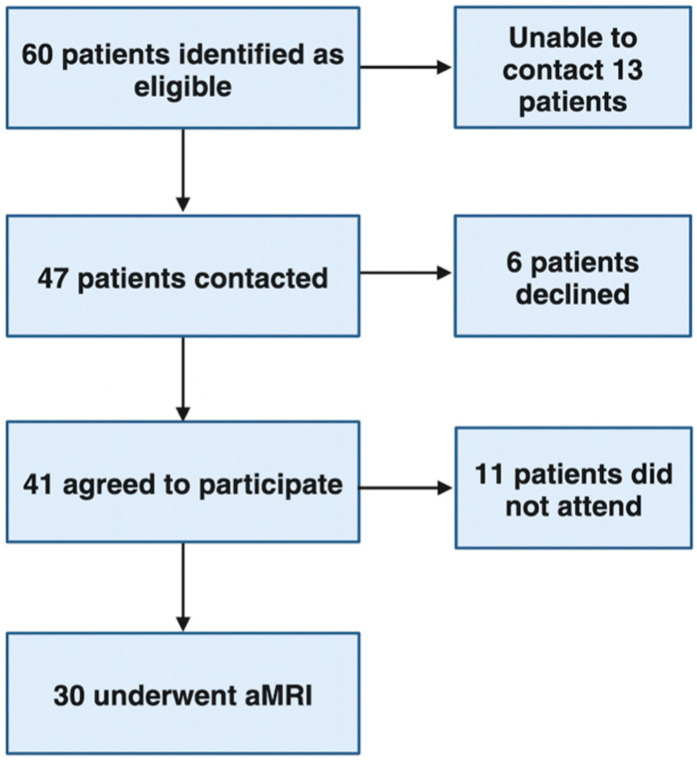
Study flowchart outlining recruitment, exclusion and withdrawal. aMRI detects lesions missed in US surveillance.

Sixty patients were identified as eligible for the study (Figure 2). Forty-seven patients were contacted by telephone and offered an aMRI. Six patients declined to participate, citing poor health, claustrophobia and multiple upcoming hospital appointments. A further 11 patients who initially agreed to participate did not attend the aMRI. Reasons for non-attendance included scheduling difficulties (10 patients) and claustrophobia (1 patient). The demographics of the study population are given in Table 1. The majority of patients were overweight (BMI > 23 in Asian participants and >25 in all other ethnicities) (93.3%), with 50% being obese (BMI > 30) and 20% severely obese (BMI ≥ 40). A total of 90% of the patients had cirrhosis, with 57% Child–Pugh A and 10% Child–Pugh B. Viral hepatitis was the most common liver disease aetiology (43.3%), with alcohol-related liver disease and NAFLD seen in 20.0% and 13.3% of patients, respectively.

In terms of US quality, 20% were reported as LI-RADS A; 40% were B, and 40% were C. All patients tolerated aMRI and completed the T2WI and DWI protocol. The median time to complete aMRI was 26 min (interquartile range: 23–28 min).

**Figure 3:**
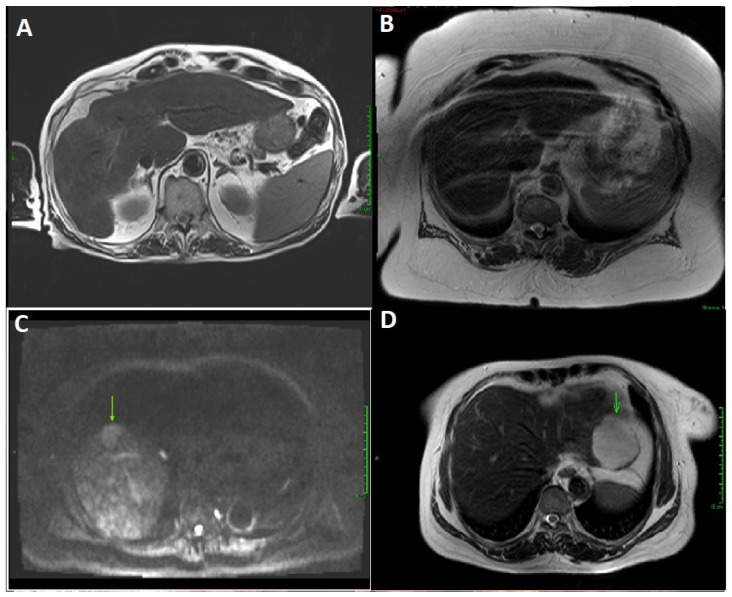
Abbreviated MRI images from study participants. (**A**). Satisfactory quality abbreviated MRI in a patient with ArLD cirrhosis, with no lesion detected. (**B**). Insufficient quality abbreviated MRI due to breathing artefact in patient with body mass index > 55. (**C**). Hepatocellular carcinoma (arrow) in a patient with cirrhosis, with no lesion detected on surveillance ultrasound. (**D**). Haemangioma (arrow) in a patient, with no lesion detected on ultrasound.

Through formal reporting by an expert liver radiologist, 28 aMRI (93.3%) scans were evaluated as being of satisfactory quality for HCC surveillance (Figure 3A). Two scans (6.7%) were deemed to be of inadequate quality for HCC surveillance due to the presence of a major breathing artefact and increased body habitus (BMI > 55), impairing visualisation.

Six patients (20%) had a hepatic abnormality detected with aMRI and underwent further contrast imaging for the characterisation of hepatic lesions. Following contrast MRI, one lesion was confirmed as HCC (Figure 3C), one lesion as haemangioma (Figure 3D), and four suspected lesions were not seen on contrast imaging or were too small to be of clinical significance.

### 3.2. Visualisation Quality Scores and Inter-Observer Agreement

The visualisation quality scores for each radiologist are shown in Table 2. Coverage of the liver was rated to be of sufficient quality by all three radiologists in 93.3%, 100% and 90.0% of scans, with a high inter-observer agreement (κ = 0.78; 95% confidence interval (CI): 0.60–0.96). Vessel differentiation (73.3%, 93.3% and 76.7%; κ = 0.60; 95% CI: 0.38–0.82) and parenchymal definition (80.0%, 60.0% and 67.8%; κ = 0.47; 95% CI: 0.23–0.70) were rated to be of sufficient quality in the majority of aMRI scans. There was a large variation in the quality scores for the signal-to-noise ratio (63.3%, 33.3% and 47.8%; κ = 0.51; 95% CI: 0.28–0.74) and uniformity of liver appearance (23.3%, 10.0% and 70.0%; κ = −0.11; 95% CI: −0.29–0.07) between radiologists.

Total number of patients given a score of 1 for each visualisation quality parameter, n, and percentage of total scans, %, are shown for individual radiologists. The mean for each visualisation quality parameter for all three radiologists and % out of total scans is shown. The number of scans rated as “acceptable” by each radiologist, with the % and mean, is shown. Randolph’s free-marginal multirater kappa, k, and 95% confidence interval for the visualisation quality parameter scores of the three radiologists are shown.

## 4. Discussion

This is the first prospective study investigating the use of non-contrast aMRI for HCC surveillance in patients with inadequate ultrasound visualisation. We found that 93% of aMRI scans were of sufficient quality in a real-world surveillance cohort of predominantly overweight and obese patients. aMRI scans had sufficient liver coverage and parenchymal definition and allowed for adequate vessel differentiation. The aMRI protocol identified two hepatic abnormalities, including one patient with HCC, which had been missed on surveillance US. Our study presents novel work, demonstrating that attenuated non-contrast MRI is a feasible modality of HCC surveillance in patients with inadequate US studies.

US is safe, non-invasive and inexpensive and, thus, favourable for HCC surveillance [24]. However, up to one-fifth of patients may have inadequate visualisation with US [25]. Inadequate visualisation can significantly reduce HCC detection, with an US LI-RADS visualisation score VIS-C associated with a sensitivity of 27% for detecting HCC [26]. Multiple factors can impact the US visualisation quality and HCC detection, including operator experience [27], cancer stage [6] and patient characteristics [9]. In particular, US visualisation is limited in patients with NAFLD due to increased body habitus, hepatitis steatosis and advanced cirrhosis [9]. A retrospective study of 167 patients who had undergone transplant for HCC showed that increased body habitus is associated with reduced HCC detection using US, with a marked drop in sensitivity from 77% to 21% in patients with a BMI ≥ 30 compared to those with a BMI < 30 [28]. Furthermore, an Italian study of 1170 patients undergoing US surveillance reported that a BMI greater than 25 was associated with failure to detect early-stage HCC in 109 patients [29].

Increased lipid deposition in liver tissue increases US beam scattering, creating a hyperechoic liver appearance in hepatic steatosis. This results in the attenuation of US signals, diminishing the ability to discriminate structures and lesions within the liver parenchyma [30]. The majority (97%) of patients in our current study were overweight, with 50% being obese and 20% with severe obesity. Importantly, we observed that an increase in BMI was not just associated with NAFLD but affected the majority of the cohort study regardless of aetiology, reflecting global rates of obesity [8]. Our non-contrast aMRI protocol was able to adequately visualise the whole liver anatomy and parenchymal changes in most of these patients. Therefore, in patients with previously inadequate US visualisation, aMRI could be used as the surveillance imaging modality as an alternative to repeated US and/or dynamic contrast-enhanced CT imaging/MRI.

Late arterial-phase hyperenhancement followed by portal venous-phase washout is the gold standard for HCC diagnosis using DCE-MRI/CT [31]. Though surveillance DCE-MRI has been shown to be superior to US for HCC detection [14], these protocols are time consuming, expensive and associated with radiation- and contrast-related toxicity. Thus, DCE-MRI is impractical as a modality for community surveillance. Abbreviated MRI utilises a reduced number of chosen sequences to maximise HCC detection whilst minimising the scanning time, cost and contrast use. Previous studies have applied simulated aMRI protocols retrospectively to cohorts scanned using DCE-MRI [13,15,17,18,19,32,33]. Different aMRI protocols have been proposed, utilising contrast and non-contrast phases. Non-contrast protocols are less expensive and non-invasive and, thus, more appealing for large-scale HCC surveillance. Non-contrast protocols have employed T1WI, T2WI and DWI [13,17,18,19,33]. The sensitivity of non-contrast aMRI protocols retrospectively simulated from full MRI protocols varies between 61.5% and 86.3% [13,18,19,33]. aMRI protocols utilising contrast have also been investigated, ranging from a single contrast phase to dynamic sequences [15,17,32,33]. Park et al. retrospectively simulated an abbreviated T2WI, DWI and hepatobiliary phase protocol in patients undergoing three cycles of six monthly surveillance scans [17]. This abbreviated protocol had a significantly higher sensitivity in detecting HCC compared to US (86.0% vs. 27.9%; *p* < 0.001), with a comparable specificity (95.6% vs. 96.3%; *p* = 0.59). A meta-analysis of 15 studies using different aMRI techniques found that contrast protocols had a pooled sensitivity and specificity of 87.0% and 94.0%, comparable to non-contrast protocols [34]. Similar to non-contrast aMRI, the majority of these studies apply simulated protocols from DCE-MRI.

Only a limited number of studies have prospectively performed aMRI for surveillance [35,36]. Huang et al. compared the visualisation qualities of US and aMRI in 54 patients with NAFLD [35]. Contrast aMRI was able to visualise > 90% of the liver in 97.9% of patients, supporting our findings. Brunsing et al. showed, in 330 patients, that gadoxetate-enhanced aMRI was diagnostically adequate in 94.2% of patients, with a sensitivity of 92.0% for HCC detection [35,36]. These studies demonstrate that aMRI can be adopted for HCC surveillance, though both utilise pre-scan contrast injection. A strength of our aMRI protocol is not requiring cannulation and contrast injection, limiting the workload and time burden on the radiology department.

There are increasing data supporting the use of MRI in populations with a high risk of HCC: a study of patients with high-risk (>3% annual risk of HCC) cirrhosis in France [37] found that contrast MRI was five times more likely than US to detect BCLC stage 0 HCC and was cost effective. These findings are supported by another study in Korea, with the authors concluding that MRI may be more cost effective than US in high-risk viral cirrhosis [38]. Markov modelling demonstrated that the most cost-effective surveillance strategy for all patients with cirrhosis was aMRI when assuming real-world compliance rates and considering inconclusive imaging [39]. Of interest, the authors noted that the cost of aMRI is directly proportional to the duration of the scan and suggest that an aMRI duration of less than 10 min would be cost effective, whereas longer scans may not be. Previous studies using simulated aMRI from contrast MRI has reported theoretical times of 5.5 to 7.0 min [13,17]. The median time of 26 min to perform aMRI in our study is more representative of real-time applications compared to these simulated studies [13,15,17,18,19,32,33].

Given that full DCE-MRI takes a minimum of 40 min [40], shorter aMRI protocols are likely to be more cost effective for HCC surveillance in patients at a high risk of inadequate visualisation with US.

Previous studies have focused on using aMRI as a first-line surveillance modality in general patient cohorts. However, no previous aMRI studies have assessed its utility in a more challenging patient cohort with previously inadequate visualisation on ultrasonography. Currently, there is no robust guidance for ongoing surveillance strategies for patients with inadequate hepatic visualisation on surveillance US, with options including continued US surveillance, dynamic-contrast enhanced CT or dynamic-contrast enhanced MRI. Poor ultrasound visualisation due to inadequate echogenic windows and macronodular parenchyma is associated with increased surveillance failure [26,41], and thus, persisting with US surveillance in patients with previous inadequate visualisation is likely to lead to repeated insufficient imaging. The use of dynamic-contrast enhanced CT imaging and MRI for regular surveillance is associated with significant costs in addition to cumulative radiation and contrast exposure [42,43]. Both EASL and AASLD advise that MRI/CT could be used in patients who have had inadequate ultrasonography, but state that due to their risk and cost, their use in long-term surveillance is highly debatable [2,4]. The non-contrast aMRI protocol utilised in our study offers a less expensive and more practical ongoing surveillance modality compared to dynamic-contrast enhanced imaging for patients with inadequate ultrasound visualisation. Our current study demonstrates that aMRI is feasible as an HCC surveillance strategy in patients with previous inadequate visualisation on ultrasound, in particular those with an elevated BMI. Thus, aMRI can be utilised as an alternative second-line surveillance imaging modality in patients with previously inadequate or at a high risk of inadequate ultrasound surveillance.

Our study has several strengths. To date, this is the first real-time prospective study utilising non-contrast aMRI for HCC surveillance compared to previous studies, which have retrospectively applied simulated aMRI sequences from patients undergoing dynamic contrast-enhanced MRIs. The median time of 26 min to perform aMRI in our study is representative of real-time applications compared to the theoretical times proposed in the literature. The quality of the visualisation parameters was evaluated by three blinded, independent radiologists, confirming the performance of the aMRI protocol. As this is a prospective study, the patient withdrawal and non-attendance for surveillance scans is representative of real clinical practice.

Our study has limitations. All aMRI outcomes were reported by an experienced liver radiologist from a single centre. To further understand its widespread use and feasibility for surveillance, the interpretation of aMRI by general radiologists, across different centres, will need to be determined in future studies. Though US images were retrospectively reviewed by an experienced US radiologist, a formal, real-time assessment of the scan quality could not be conducted. This limits the direct comparison between aMRI and US visualisation. In this study, patients underwent a single aMRI, representing a single six-monthly surveillance scan cycle. Subsequent prospective studies evaluating aMRI will require follow-ups with multiple surveillance cycles to determine patient acceptability, given that MRI-related anxiety and claustrophobia have been reported to be as high as 15% [44]. As only 30 patients were included in this feasibility study, the prevalence of HCC is low; hence, the true sensitivity and specificity of aMRI cannot be determined. However, we demonstrate the feasibility of non-contrast aMRI in patients with inadequate US. We observed significant non-attendance for aMRI: 24% of patients who agreed to participate and were scheduled for aMRI did not attend. However, in the context of global poor attendance at HCC surveillance (19% uptake in American cohorts [45]), our attendance rate of 76% is promising and reflects patient preference for aMRI over US as a surveillance tool [46], in addition to improved uptake with reminder phone calls [47]. Further large, randomised control studies directly comparing aMRI with US will determine the accuracy of aMRI for surveillance in patients with impaired visualisation of the liver with US.

## 5. Conclusions

This is the first prospective study to demonstrate that non-contrast aMRI can be used for HCC surveillance in patients with previously inadequate visualisation on ultrasound. In our enriched cohort of overweight and obese patients with inadequate surveillance US, aMRI provides satisfactory visualisation of the liver, allowing for the detection of HCC. Further randomised control trials will elucidate the role of aMRI as an alternative surveillance imaging modality for patients with inadequate US, in lieu of more expensive dynamic-contrast enhanced imaging.

### Novelty and Impact

Ultrasound surveillance for hepatocellular carcinoma (HCC) is widely used but has limitations. We investigated the feasibility of non-contrast, abbreviated magnetic resonance imaging (aMRI) as a surveillance tool. Our research is the first prospective study of abbreviated MRI in an HCC surveillance cohort. We demonstrate that abbreviated MRI is able to identify HCC not identified using US.

## Figures and Tables

**Table 1:** Patient characteristics at baseline.

Characteristic	Value
**Male sex n (%)**	25 (83.3%)
**Mean age, range, ± SD**	57 (36–73) ± 8.9 years
**Ethnicity n (%)**	
White	12 (40.0%)
Asian	5 (16.7%)
Black	5 (16.7%)
Mixed	1 (3.3%)
Other	7 (23.3%)
**Median weight, range**	90 kg (66–146)
**Median BMI, range**	29.6 (23–55)
Overweight (BMI ≥ 25) n (%)	28 (93.3%)
Obese (BMI ≥ 30) n (%)	15 (50.0%)
Severe obesity (BMI ≥ 40) n (%)	6 (20.0%)
**Liver disease aetiology n (%)**	
Viral hepatitis	13 (43.3%)
NAFLD	4 (13.3%)
ArLD	6 (20.0%)
Autoimmune liver disease	4 (13.3%)
Other/mixed aetiology	3 (10.0%)
**Comorbidity** *	
No significant comorbidity	20 (67%)
Significant comorbidity	10 (33%)
**Child-Pugh Score median, range**	
Child–Pugh A	17 (57%)
Child–Pugh B	3 (10%)
Non-cirrhotic	10 (33%)

SD—standard deviation; BMI—body mass index; NAFLD—non-alcoholic fatty liver disease; ArLD—alcohol-related liver disease. * Significant comorbidity: cardiac disease; pulmonary disease; chronic kidney disease; diabetes; cancer.

**Table 2:** Visualisation quality parameter scores by three independent radiologists for thirty attenuated MRI hepatocellular carcinoma surveillance scans with inter-observer agreements.

Visualisation Quality Parameter	Radiologist 1 n (%)	Radiologist 2 n (%)	Radiologist 3 n (%)	Radiologist Average n (%)	Inter-Observer Agreement κ (95% CI)
Parenchymal definition	24 (80.0%)	18 (60.0%)	19 (63.3%)	20.3 (67.8%)	0.47 (0.23, 0.70)
Vessel differentiation	22 (73.3%)	28 (93.3%)	23 (76.7%)	24.3 (81.1%)	0.60 (0.38, 0.82)
Coverage and volume of liver	28 (93.3%)	30 (100%)	27 (90.0%)	28.3 (94.4%)	0.78 (0.60, 0.96)
Uniformity of liver appearance	7 (23.3%)	3 (10.0%)	21 (70.0%)	10.3 (34.4%)	0.11 (−0.12, 0.34)
Signal-to-noise ratio	19 (63.3%)	10 (33.3%)	14 (46.7%)	14.3 (47.8%)	0.51 (0.28, 0.74)

## Data Availability

All data and analysis materials will be available upon request to the corresponding author, Professor Rohini Sharma (r.sharma@imperial.ac.uk).
